# Observations and Recommendations From the Mobilizing Action Toward Community Health (MATCH) Expert Meeting

**Published:** 2010-10-15

**Authors:** David A. Kindig, Bridget C. Booske, Kirstin Q. Siemering, Patrick L. Remington, Brenda L. Henry

**Affiliations:** University of Wisconsin School of Medicine and Public Health, Population Health Institute; University of Wisconsin, Madison, Wisconsin; University of Wisconsin, Madison, Wisconsin; University of Wisconsin, Madison, Wisconsin; Robert Wood Johnson Foundation, Princeton, New Jersey

## Introduction

In October 2009, authors, staff, and guest experts from the Mobilizing Action Toward Community Health (MATCH) project and the Robert Wood Johnson Foundation, the project's funder, met in Madison, Wisconsin to discuss metrics, incentives, and partnerships for population health improvement. Their essays were published in this and the previous 2 issues of *Preventing Chronic Disease* (www.cdc.gov/pcd/issues/2010/jul/toc.htm and www.cdc.gov/pcd/issues/2010/sep/toc.htm). The plenary and small-group discussions were provocative and wide ranging. The purpose of this commentary is to 1) summarize key themes from the essays and meeting discussion and 2) present recommendations for future practice and research regarding metrics, incentives, and partnerships to improve population health.

## Discussion Themes

### Metrics

Bilheimer and Pestronk presented commentaries on the metrics essays (1,2). Meeting participants identified challenges related to population health metrics. They recognized that the usefulness, reliability, and validity of metrics are often compromised by limitations in available data. Examples of these complicating factors include sparsely populated geographic areas, challenges with survey methods (such as random-digit dialing in a cell phone era), and the choice of unit of analysis.

Geopolitical areas such as counties or states are often used because they are the focus of much of the available data, but these areas do not necessarily reflect population health *market areas* where programs and policies are implemented to improve health outcomes. Data intricacies add complexity to analyses — as is illustrated by the fact that different health determinants operate in different geographic areas (eg, school nutrition policies are local, air quality policies are regional, and Medicare policies are national).

Participants agreed that the population health field needs revised metrics to address various goals.

- **Population-based metrics to monitor changes in population health.** Most measures of population health (eg, those used in the *County Health Rankings*) are used to measure differences between geographic areas and often combine several years of data to increase the precision of the estimates (3). More precise metrics are needed to monitor trends over time and show changes over short time frames in response to local-level changes in programs and policies.
- **Standard measures of health disparities within communities.** Most measures of population health can demonstrate disparities *between* geographic areas (eg, the *County Health Rankings*), but more attention needs to be focused on disparities *within* communities by using different disparity domains such as race/ethnicity and socioeconomic factors.
- **Metrics that can be easily understood by the public and policy makers.** Many metrics that reflect the health of a population (eg, age-adjusted death rates) are difficult to communicate to the public or to policy makers. Approaches such as dashboards (which use graphics resembling gauges and dial-type indicators) or rankings can improve communication and awareness or generate action among targeted and broad audiences.

One participant suggested that, "A good measure makes you feel responsible for taking action." Another noted that measurement is an assertion of responsibility; population health should be measured at appropriate levels so that disparities are not masked and should include a wide set of measures so that governments and other relevant entities (eg, business, education, transportation) can take responsibility. Participants also preferred an interpretable logic model so that audiences understand the choice of metrics: Why is each measure important and what can be done about it? What are the pathways, how can they be influenced, and at which levels?

### Incentives

McGinnis and Lewis provided commentaries on the essays that examined the use of incentives to improve population health (4,5). Meeting participants discussed the process of creating incentives to improve population health, and how incentives should link to measures of desired outcomes. Although much of the discussion focused on financial incentives, participants also addressed nonfinancial incentives such as political gain or professional recognition. For example, it was noted that California's quality improvement in health care was largely driven by public reporting and information sharing. The desire to achieve such recognition on published lists may fuel innovative and sustained change.

As a result of current private and public fiscal instabilities, perhaps financial incentives should be directed toward identifying new resources or redirecting existing ones. Would resources be one-time grants from government and foundations, or would they be built into formulas like the community benefit tax rules to ensure the long-term investments that would be needed?

Participants noted that incentives must be linked to individual or organizational self-interests to affect change. Unfortunately, no consensus exists on which specific incentives best motivate individuals, organizations, and sectors and how factors such as values, ideology, and beliefs affect the power of incentives at all levels. We need to better understand how incentives have been used both successfully and unsuccessfully in education, welfare, and other social systems. Although government entities generally adopt a directive (ie, top-down) approach to incentives, incentives can also be effectively initiated from the bottom up, in which individuals and investors decide how and where to direct their resources.

### Partnerships

Shortell and Bailey provided commentaries on the population health partnership essays (6,7). Participants observed that partnerships are anything but one-size-fits-all; they may be characterized across a spectrum of collaboration ranging from cooperation to integration. Participants raised various issues on the partnership theme.

- **Identifying best practices in community partnerships.** Given the wide variability in partnership structure and function, participants wanted to know if best-practice processes can be identified that apply across the board (such as with respect to capacity building and strategic planning). For example, do partnerships require a minimum level of formality to effectively share power and drive action? What factors cause partnerships to have a more formal structure and function?
- **Sustaining partnerships.** Participants wanted to know more about how partnerships earn credibility and legitimacy over time and how community institutions can prevent or resolve conflict that could hinder strong cross-sectoral collaborations. For example, how are costs and benefits evaluated from the perspective of prospective partners (transaction costs of formation vs potential for synergy once established)?
- **Balancing competing priorities.** Participants asked how partnerships could balance core competence (what they accomplish in an absolute sense based on available expertise, skills, and resources) with comparative advantage (what they can accomplish in a relative sense based on what they do better than others). In addition, they wanted to know the degree to which having a population health agenda shared (overtly or not) by sectors outside health, what might motivate nonhealth sectors to come to the table, and whether a multisectoral investment logic model could be developed for all partners.

Participants noted that there is no substitute for effective leadership throughout all phases of partnership. Without questioning the potential of partnerships, they challenged the notion that partnerships are necessary for improved population health. Participants did not doubt that multiple sectors should be engaged in efforts to address the multiple determinants of health, but several questioned whether improvement actually requires cross-sector work. In other words, is it possible to effect substantial change through focused intrasector activity? One possible response is that the nature of the task at hand often determines the level of cross-sectoral coordination required: solving bigger problems is likely to require more interdependence, particularly the sharing of resources.

## Recommendations for Practitioners

In breakout groups, participants identified 3 opportunities for future work among practitioners: increasing investments in multiple determinants of population health, establishing service bureaus to provide technical assistance, and establishing an award for population health improvement.

### Increase investments in the multiple determinants of population health

Discussion regarding investments centered on aligning resources and incentives to drive investment in programs and policies that will improve health outcomes and reduce disparities. Suggestions included developing investment pools similar to those being tried by the California Endowment. The California Endowment is using funds for intervention via multisectoral partnerships or enhancing naturally occurring multisectoral initiatives. Such interventions should require investments in the multiple determinants of health, including income and educational policies and the built environment. To increase the likelihood of success, meeting participants recommended focusing investments in places where some partnership activity already exists and where infrastructure is in place.

This recommendation has several challenges. For example, how should investments be balanced between communities with the need and those with the highest likelihood of success? Also, who will provide the necessary resources? Although government, foundations, and business and community investments are reasonable sources, discussion also focused on other sources that might be more dependable and permanent, such as savings captured from waste on unnecessary health care. Some discussion focused on the policy proposals for accountable care organizations (ACOs) in Medicare, which could generate savings for high-quality and low-cost care. Instead of only sharing savings with providers and payers, a portion could be used as a community health dividend. The Vermont Blueprint for Health (8) has used such an approach, and leaders in Minnesota have called for nesting ACOs in accountable health communities. Participants also suggested that the community benefit definition used by the Internal Revenue Service be expanded to include the value of hospital investment in local population health improvement that goes beyond charity care. The 2010 Patient Protection and Affordable Care Law (Pub L No. 111-148) represents a step in the right direction by requiring nonprofit hospitals to conduct a needs assessment in consultation with the communities they serve at least every 3 years.

### Establish technical assistance service bureaus

Many participants noted the lack of community capacity and expertise for population health improvement activities such as using metrics to leverage investment and create effective partnerships. Local or virtual technical assistance could be provided to use data for health improvement, identify evidence-based policies and programs, create processes to identify and implement local interventions, set cost-effective priorities, and help community partners recognize the need for cross-sector collaboration for health improvement. For example, public and private funders could be more prescriptive in providing a menu of evidence-based programs and interventions.

### Establish a population health improvement award

The idea of a Baldrige-like (9) annual prize for communities excelling in improving population health through creative use of incentives, metrics, and partnerships was proposed. Participants noted that recognition of improvement should take account of change over time and achievement or accomplishment at a point in time.

## Recommendations for Researchers

Participants identified some major research needs and opportunities that could move understanding and action forward in the population health field. They included examining causal relationships between determinants of health, increasing understanding of population health incentives, and increasing understanding of population health partnerships.

### Examine causal relationships between determinants of health

Participants recommended that funders should support research to examine the cost-effectiveness of addressing different determinant categories and also specific programs and policies. This research should also address secondary health effects of nonhealth policies, for example by expanding the scope of comparative effectiveness research to include determinants of health beyond health care. In addition, research should be conducted to improve metrics that can monitor changes in population health and to propose ways to balance incentives for population health improvement. Researchers should also develop more robust disparity measures for health outcomes and health determinants.

### Increase understanding of population health incentives

Researchers should develop an expanded multisector population health model so that leaders understand their roles, responsibilities, and most cost-effective actions for population health improvement within and outside of their own sectors. Research on these investments should also determine what cross-sectoral financial and policy investment at the community level has been successful in improving health. The information can then be used to develop local (ie, substate) data sets for understanding these relationships.

Researchers should also determine the advantages and disadvantages of applying incentives at different levels of aggregation (ie, individual vs community vs organization), the advantages and disadvantages of using bundled or unbundled metrics for applying incentives, and how to avoid poor performers receiving penalties when they need resources to improve. Finally, research should examine the scope of potential nonmonetary and monetary incentives for population health in the United States and abroad.

### Increase understanding of population health partnerships

Research should be conducted to better understand public- and private-sector leaders' attitudes toward population health improvement and tradeoffs. Where do population health improvement and disparity reduction (in general) fall on their priority list? Who (outside of the health community) is paying attention?

Research on partnerships should also identify the characteristics of effective partnerships. How can they be developed, expanded, and sustained? Are partnerships necessary for population health improvement, or can sectors operate effectively alone? Which organizations are candidates to be integrators across the population health model?

## Conclusion

The 2009 MATCH expert meeting generated thoughtful and stimulating discussion around the essays presented in this and the previous 2 issues of *Preventing Chronic Disease*. Far more questions were asked at the meeting than answered. Through facilitated dialogue, participants offered wide-ranging ideas and insights in the areas of metrics, incentives, and partnerships. The meeting provided little time or space for many details; the format necessitated input in rather broad brushstrokes toward the goal of building consensus for practice and research priorities. As the essays and commentaries in this series attest, improving population health will require effort on many fronts; no single track to success exists. Whereas the challenges are substantial, the ideas shared here should be reflected on, refined, expanded, and hopefully pursued through empirical and applied efforts to improve population health.
